# Phthisis

**Published:** 1855-07

**Authors:** 


*Phthisis.—Night Sweats of.*—Give the following draught at bedtime:

℞ Acid gallic, gr. vij;

Morphiæ acet., gr. $\frac{1}{8}$;

Alcohol, q. s. (a few drops);

Syr. tolutan, ʒss;

Aquæ, ℥j.

The night-pill of the Brompton Hospital is as follows:

℞ Acid gallic, gr. v;
Morphiæ hydrochlor. gr. $\frac{1}{8}$;
Mist. acaciæ. q. s. Ft. pil. ij. (Mr. Hutchinson.)

Give gallic or acetic acid. Dip the night dress in sea-water, or salt and water, and dry it before using. But the best remedy is four grains of oxide of zinc at bedtime, combined with a little henbane or hemlock.

*Cough of.*—Mix one part of chloroform with three-parts of spirits of wine, and let the patient inhale when necessary, but with caution, and under medical direction. The inhalation of camphorated spirit is often sufficient, or even the vapor of hot water, or infusion of hops. Sometimes frequent deglutition, as the swallowing a little oil, will relieve the cough. Sometimes four minims of tincture of aconite is a good palliative.

*Profuse Expectoration of.*—To check this give creasote, pyro-acetic spirit, infusion of pitch, or balsam of tolu; but by far the best remedy is petroleum or Barbadoes tar, which often moderates the cough and expectoration remarkably.—*Braithwaite's Retros.*

